# The crystal structure of the tetrameric human vasohibin-1–SVBP complex reveals a variable arm region within the structural core

**DOI:** 10.1107/S2059798320011298

**Published:** 2020-09-16

**Authors:** Akihito Ikeda, Seia Urata, Tadashi Ando, Yasuhiro Suzuki, Yasufumi Sato, Tatsuya Nishino

**Affiliations:** a Department of Biological Science and Technology, Tokyo University of Science, 6-3-1 Niijyuku, Katsushika-ku, Tokyo 125-8585, Japan; b Department of Applied Electronics, Faculty of Industrial Science and Technology, Tokyo University of Science, 6-3-1 Niijyuku, Katsushika-ku, Tokyo 125-8585, Japan; c Department of Vascular Biology, Institute of Development, Aging and Cancer, Tohoku University, 6-6-10 Aramaki Aza Aoba, Aoba-ku, Sendai, Miyagi 980-8575, Japan; d New Industry Creation Hatchery Center, Tohoku University, 6-6-10 Aramaki Aza Aoba, Aoba-ku, Sendai, Miyagi 980-8575, Japan

**Keywords:** vasohibin, small vasohibin-binding protein, protein complex, X-ray crystal structure, MD simulations, microtubule modification, VASH1–SVBP complex

## Abstract

Vasohibins regulate angiogenesis, tumor growth, metastasis and neuronal differentiation. They form a complex with small vasohibin-binding protein (SVBP) and show tubulin tyrosine carboxypeptidase activity. Recent crystal structure determinations of vasohibin–SVBP complexes have provided a molecular basis for complex formation, substrate binding and catalytic activity. However, the regulatory mechanism and dynamics of the complex remain elusive. Here, the crystal structure of the VASH1–SVBP complex and a molecular-dynamics simulation study are reported. The overall structure of the complex was similar to previously reported structures. Importantly, however, the structure revealed a domain-swapped heterotetramer that was formed between twofold symmetry-related molecules. This heterotetramerization was stabilized by the mutual exchange of ten conserved N-terminal residues from the VASH1 structural core, which was intramolecular in other structures. Interestingly, a comparison of this region with previously reported structures revealed that the patterns of hydrogen bonding and hydrophobic interactions vary. In the molecular-dynamics simulations, differences were found between the heterotetramer and heterodimer, where the fluctuation of the N-terminal region in the heterotetramer was suppressed. Thus, heterotetramer formation and flexibility of the N-terminal region may be important for enzyme activity and regulation.

## Abstract 2

Vasohibin-1 and small vasohibin-binding protein (SVBP) form an intermolecular heterotetramer in the crystal. The heterotetramer was stabilized by exchange of the conserved N-terminal region.

## 1. Introduction   

Vasohibin was first discovered as a factor involved in angiogenesis and is widely conserved among vertebrate species. Two vasohibin paralogs, vasohibin-1 (VASH1) and vasohibin-2 (VASH2), exist in most species, where the former inhibits and the latter promotes neoangiogenesis (reviewed in Sato, 2013 ▸). Vasohibins form a complex with small vasohibin-binding protein (SVBP) and are processed and secreted through the noncanonical pathway. Vasohibins and SVBP have recently been found to be involved in the development of neurons and normal basement-membrane formation in the renal corpuscles (Pagnamenta *et al.*, 2019 ▸; reviewed in Tanabe *et al.*, 2018 ▸). In this pathway, vasohibin participates in the post-translational modification of tubulin, which is known to control neuron differentiation. Both the VASH1–SVBP and VASH2–SVBP complexes function as a tubulin carboxypeptidase to cleave the terminal tyrosine residue, and thus the difference between the two paralogs remains elusive.

Proteases are known to be regulated in multiple stages from their expression to the functional state. For example, serine proteases such as trypsin are initially expressed in an inactive zymogen form (trypsinogen) and are subsequently cleaved for activation. Calcium binds tightly to trypsin and stabilizes the protease. Other proteases such as pepsin, gastricsin and renin from the aspartic protease family are also expressed as zymogens and are autoinhibited through a prosegment that precedes the protease domain. The prosegment binds to the protease domain and keeps it inactivated until proteolysis (Dunn, 2002 ▸). Vasohibins form a complex with SVBP and complex formation is required for secretion (Suzuki *et al.*, 2010 ▸). Sequence analysis of vasohibins identified that they belong to the transglutaminase-like cysteine proteases, with conserved catalytic residues and a putative Ca^2+^-binding site (Sanchez-Pulido & Ponting, 2016 ▸). Crystal structures of the vasohibin–SVBP complex revealed that the catalytic site is indeed similar to that of transglutaminase (Adamopoulos *et al.*, 2019 ▸; Li *et al.*, 2019 ▸; Liao *et al.*, 2019 ▸; Wang *et al.*, 2019 ▸; Zhou *et al.*, 2019 ▸; Liu *et al.*, 2019 ▸). SVBP binds to the N-terminal structural core region of vasohibin and stabilizes the complex. The structures of complexes with tubulin peptides or inhibitors revealed how vasohibin recognizes substrates (Li *et al.*, 2019 ▸; Liao *et al.*, 2019 ▸; Wang *et al.*, 2019 ▸; Zhou *et al.*, 2019 ▸). However, little is known about the dynamics and regulation of the vasohibin complex.

Vasohibins contain less conserved N-terminal and C-terminal regions which are predicted to be disordered and flexible. Previous studies have found that VASH1 is processed at Arg29 and Arg76 (Sonoda *et al.*, 2006 ▸). Cleavage at Arg29 can be explained by the disordered structure. In contrast, Arg76 is part of the SVBP-binding helix, and processing at this residue suggests some dynamic property, but its exact nature remains elusive. We set out to investigate the nature of this region and the structural dynamics of the VASH1–SVBP complex. Surprisingly, we found unexpected heterotetramerization and flexibility within the protease domain which had not been seen in other structures. We performed molecular-dynamics (MD) simulations of the heterotetramer and heterodimer in order to compare their conformational flexibility.

## 2. Materials and methods   

### 2.1. DNA cloning and protein preparation   

Human VASH1 consists of 364 amino acids, and its molecular weight is 40 825 Da. Genes for the human VASH1 core (VASH1c) region (amino acids 56–310) and human SVBP (amino acids 2–66) were amplified from pcDNA3.1 and expression vectors (Kadonosono *et al.*, 2017 ▸). The PCR fragments were cloned into a modified pMalc2x vector (New England Biolabs). To obtain a soluble stoichiometric VASH1–SVBP complex, we created a fusion protein comprising the N-terminal MBP tag, a hexahistidine tag, 5× TEV protease-recognition sequence, VASH1 (amino acids 56–310), 2× TEV protease-recognition sites and SVBP (amino acids 2–66).

For expression of the protein complex, the plasmid (CSBP255) was transformed into One Shot BL21 Star (DE3) pLysS *Escherichia coli* competent cells (Invitrogen), which were selected on an LB plate with 100 µg ml^−1^ ampicillin. The transformed bacterial colonies were inoculated in LB medium with 100 µg ml^−1^ ampicillin and were cultured at 30°C until the OD_600 nm_ reached 0.5. Protein expression was induced by the addition of 0.2 m*M* IPTG and incubation for a further 48 h. The bacterial cells were harvested by centrifugation. The bacterial pellet was resuspended in buffer A500 (10 m*M* Tris–HCl, 500 m*M* NaCl) and was sonicated using a Sonicator XL2020 ultrasonic homogenizer (Misonix) on ice. The lysate was centrifuged at 12 000*g* for 15 min. The cleared lysate was applied onto a HisTrap FF column (GE Healthcare) and was eluted with a linear gradient of elution buffer (10 m*M* Tris–HCl, 500 m*M* NaCl, 250 m*M* imidazole). The eluate was diluted to 100 m*M* NaCl with buffer A0 (10 m*M* Tris–HCl pH 8.0), applied onto an SP Sepharose column (GE Healthcare) and eluted with a salt-concentration gradient using buffer A1000 (10 m*M* Tris–HCl pH 8.0, 1 *M* NaCl). The complex was cleaved using a homemade TEV protease. The cleaved product was diluted with buffer A0 and further purified using SP Sepharose as described above, and the eluate was applied onto a Superdex 200 column (GE Healthcare) pre-equilibrated with buffer A500. The peak fraction was concentrated using an Amicon Ultra-15 centrifugal filter unit (30 kDa molecular-weight cutoff; Merck). The protein was dialyzed against 10 m*M* CHES pH 9.4.

### 2.2. Crystallization and structural determination of the VASH1–SVBP complex   

The VASH1–SVBP complex was crystallized by mixing equal amounts of protein solution (17.7 mg ml^−1^) and solutions from The JCSG Core Suite II crystallization screening kit (Qiagen). The optimal crystal was obtained using the sitting-drop vapor-diffusion method at 20°C and a condition consisting of 0.16 *M* ammonium sulfate, 0.08 *M* sodium acetate pH 4.6, 20%(*w*/*v*) PEG 4000, 20%(*v*/*v*) glycerol. Crystals were harvested in the crystallization solution and were flash-cooled under a nitrogen stream. X-ray diffraction data were collected on BL1A at the Photon Factory synchrotron facility (KEK). Diffraction data were processed by *XDS* (Kabsch, 2010 ▸). The structure was determined by molecular replacement using the *Phenix* package (Liebschner *et al.*, 2019 ▸). The coordinates of the human VASH1–SVBP complex (PDB entry 6nvq; Adamopoulos *et al.*, 2019 ▸) were used as a search model. The model was refined using iterative modeling and refinement in *Phenix* and *Coot* (Emsley *et al.*, 2010 ▸). The final model contains VASH1 (chain *A*, amino acids 56–304), SVBP (chain *B*, amino acids 26–53), four sulfate ions and 76 water molecules. Figures were prepared using *Coot*, *BIOVIA Discovery Studio* (Dassault Systèmes, Vélizy-Villacoublay, France) and *VMD* (Humphrey *et al.*, 1996 ▸).

### 2.3. MD simulation of the VASH1–SVBP complex   

X-ray crystal structures of VASH1–SVBP complexes (PDB entries 6j7b and 6ocf and the heterotetramer solved in this study; Liao *et al.*, 2019 ▸; Li *et al.*, 2019 ▸) were used for MD simulations. To prepare simulation systems of the complexes, solvents and small ligands, including an inhibitor, were removed. Using the *tleap* program in *Amber* (Case *et al.*, 2018 ▸), the complex systems were neutralized with Cl^−^ ions and solvated in a box using the TIP3P water model, where the distance between the water-box wall and the atoms of the proteins was set to 12 Å in the *x*, *y* and *z* directions. Periodic boundary conditions were applied to all simulation systems. The *Amber* ff14SB force-field parameters were used for the proteins (Maier *et al.*, 2015 ▸). The prepared systems were initially minimized first by the steepest-descent method for 1000 steps followed by the conjugate-gradient method for 1000 steps. The temperatures of the systems were then linearly increased from 1 to 310 K for 100 ps at 1 atm pressure. After an additional 100 ps MD simulation at 310 K and 1 atm for equilibration, a 100 ns MD simulation was performed to analyze the structural properties of the complexes. A cutoff distance of 12 Å was used for van der Waals and short-range electrostatic interactions, and long-range electrostatic interactions were computed using the particle-mesh Ewald summation method. A 2 fs time step was used for all MD simulations, in which all bonds containing hydrogen were constrained by the *SHAKE* algorithm (Ryckaert *et al.*, 1977 ▸). Structures and energies were sampled every 2 ps. The temperature was controlled by the Langevin thermostat (Loncharich *et al.*, 1992 ▸) with a 1.0 ps^−1^ collision frequency. The pressure was controlled by the Berendsen barostat (Berendsen *et al.*, 1984 ▸) with a pressure-relaxation time of 1.0 ps. For each VASH1–SVBP complex, three independent MD simulations were performed with different random seeds for the Langevin thermostat. For analysis, the first 10 ns of the trajectories were removed. The root-mean-square fluctuation (r.m.s.f.) of C_α_ atoms for each complex was calculated after superimposing sampled structures on the average structure. For the heterotetrameric complex, the r.m.s.f. values of chains *A* (VASH1) and its symmetric chain *D* (VASH1) were independently calculated to remove the effects of relative motion between them on the local fluctuation. All MD simulations and analyses were performed using the *Amber*18 and *AmberTools*18 packages (Case *et al.*, 2018 ▸).

### 2.4. PDB deposition   

The structure of the human VASH1c–SVBP complex has been deposited in the PDB as entry 6lpg.

## 3. Results and discussion   

### 3.1. Crystal structure of the VASH1c–SVBP complex   

**Figure 1:**
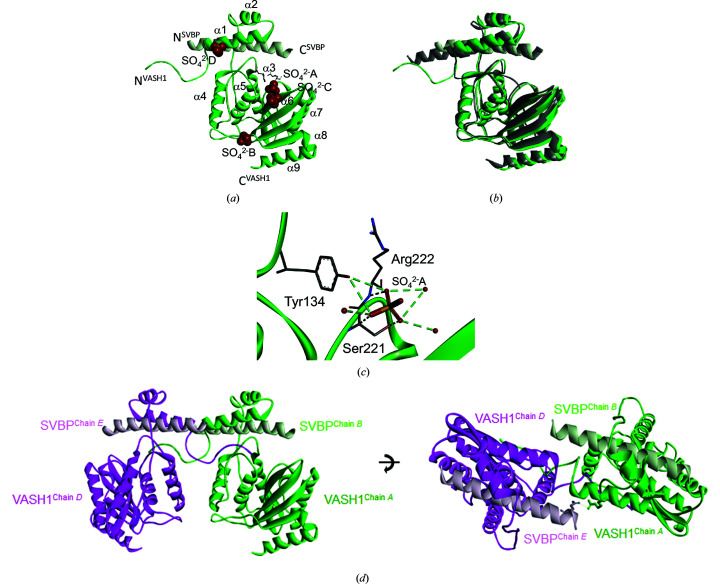
Crystal structure of the VASH1c–SVBP complex. (*a*) The VASH1c–SVBP crystal structure in the asymmetric unit. VASH1c is colored green and SVBP is colored light green. Four sulfate ions are shown as spheres and are labeled A–D. Nine α-helices of VASH1c are numbered from the N-terminus to the C-terminus. (*b*) Comparison of VASH1c–SVBP structures. The VASH1c–SVBP structure was superimposed on the reported crystal structure (PDB entry 6j7b, gray). (*c*) Sulfate ion (SO_4_^2−^A) bound in the vicinity of the catalytic triad. Three residues (Tyr134, Ser221 and Arg222) in contact with the sulfate ion are highlighted as stick models and water O atoms are shown as red spheres. (*d*) The VASH1c–SVBP heterotetramer structure in two orthogonal views. A neighboring crystallographic twofold symmetry-related molecule is shown as a ribbon representation. Chains *A* and *D* (green and magenta, respectively) correspond to VASH1c. Chains *B* and *E* (light green and pink, respectively) correspond to SVBP. Arg76 of chain *A* (VASH1c) and Glu28 of chain *E* (SVBP) that form a hydrogen bond to the symmetry-related molecule are shown as stick models.

To understand the structural basis of VASH1c–SVBP, we crystallized the purified human VASH1c–SVBP complex. The complex was crystallized in space group *P*6_1_22 and its structure was determined at 2.3 Å resolution by molecular replacement (Fig. 1 ▸ *a*, Supplementary Fig. S1, Table 1 ▸). The overall conformation of the VASH1c–SVBP dimer was highly similar to the reference structure, with a root-mean-square deviation (r.m.s.d.) of 0.42 Å (Fig. 1 ▸ *b*). In the structure, four sulfate ions were bound to the basic surface of VASH1–SVBP. Some of the reported VASH–SVBP structures contained sulfate or phosphate ions bound to the basic surface (Li *et al.*, 2019 ▸; Wang *et al.*, 2019 ▸). Interestingly, one of the sulfate ions was found in a position close to the catalytic triad and interacted with Tyr134, Ser221 and Arg222 (Fig. 1 ▸ *c*). These three amino-acid residues were important in directly recognizing the tubulin peptides and the epoY inhibitor (Liao *et al.*, 2019 ▸; Li *et al.*, 2019 ▸). Vasohibins are known to bind to the C-terminal tail region of α-tubulin, which is rich in glutamate residues. Thus, the bound sulfate ion might mimic the peptide–inhibitor interaction.

While the overall structural features were consistent with the reported structures, we noticed that the N-terminal region (amino acids 56–67) did not align well and protrudes from the VASH1c–SVBP core (Fig. 1 ▸ *b*). A comparison of our structure with previously reported structures indicated that none of the reported structures were in the same orientation. Surprisingly, the N-terminal region was inserted into the neighboring crystallographic symmetry-related VASH1c–SVBP molecule to form a heterotetramer (Fig. 1 ▸ *d*). The heterotetramer was formed by the crystallographic twofold axis; thus, the N-terminal region of the other molecule was inserted into the symmetry-related molecule.

**Figure 2:**
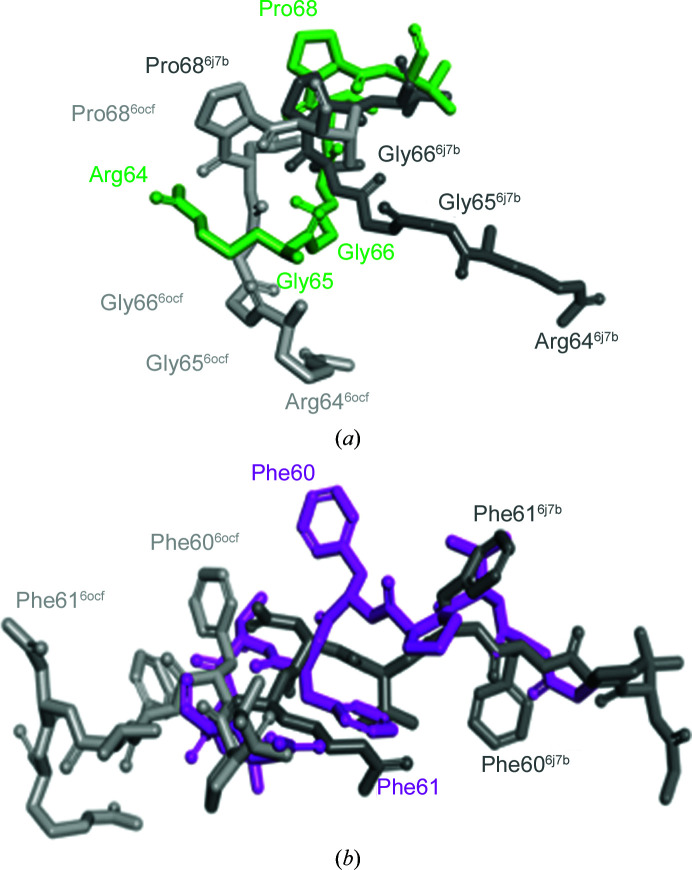
Conformations of the N-terminal region of VASH1c in three different VASH1c–SVBP complex structures. (*a*) Close-up view of the VASH1 N-­terminal region (Arg64–Pro68) shown as a colored stick model: heterotetramer (chain *A*), green; PDB entry 6j7b, gray; PDB entry 6ocf, light gray. (*b*) Close-up view of the VASH1 N-terminal region (Gly57–Arg64) shown as a colored stick model: heterotetramer (chain *D*), magenta; PDB entry 6j7b, gray; PDB entry 6ocf, light gray

The superposition of VASH1 structures revealed variation in the conformations of the N-terminal region. There were even some structures in which this region was absent. Among the structures that contain this N-terminal region, we picked two structures (PDB entries 6j7b and 6ocf) that best represent the conformational variations. Superposition of the three structures revealed that the position of Gly65 differed greatly and the side chain of Arg64 faced in opposite directions (Fig. 2 ▸ *a*). The N-terminal region preceding *cis*-Pro68 shows large conformational variations, whereas the region that follows Pro68 similarly forms an α-helix and superimposes well. When we measured the angles made by the C_α_ atoms of Glu71, Pro68 and Arg64, the heterotetramer was most extended, with an angle of 112°. The angles made by the heterodimers are more acute (95° in PDB entry 6ocf and 74° in PDB entry 6j7b), and PDB entry 6j7b shows a sharp turn. The comparison of torsion angles indicates a large difference in Gly65 and Gly66, indicating peptide flipping (Table 2 ▸). As a result of this structural variation, the region from Gly56 to Arg64 was inserted into the cleft in the VASH1–SVBP core domain (Fig. 2 ▸ *b*).

**Figure 3:**
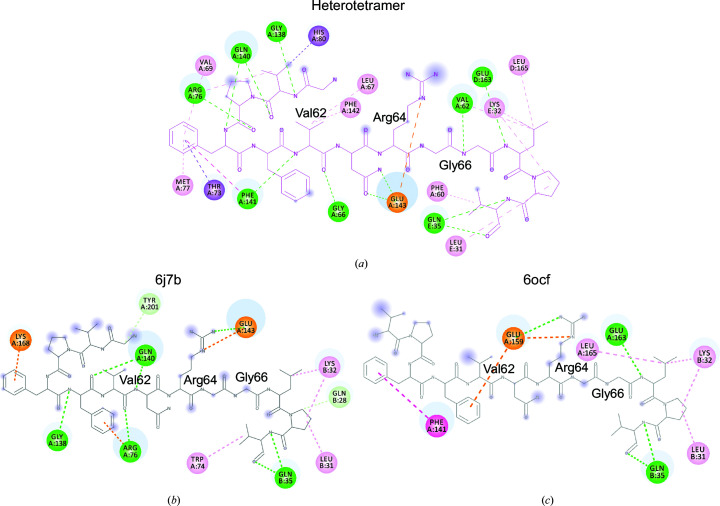
An interaction map of the VASH1c N-terminal region. (*a*) Heterotetramer. VASH1 chain *D* is shown as a structural formula colored magenta. Chains *A* and *D* are VASH1 and chain *E* is SVBP. (*b*) The PDB entry 6j7b heterodimer. VASH1 chain *A* is displayed as a structural formula colored dark gray. Chain *B* is SVBP. (*c*) The PDB entry 6ocf heterodimer. VASH1 chain *A* is displayed as a structural formula colored light gray. Chain *B* is SVBP. Intramolecular and intermolecular interactions of the N-terminal region are analyzed and shown in the 2D diagram. Interacting amino acids are shown in a circle with chain ID and residue numbers. The circles are colored according to the following scheme. Attractive charge interaction, orange; hydrogen bond, green; π–σ interaction, purple; π–π T-shaped interaction, magenta; alkyl–π-alkyl interaction, pink. The size of the blue shadow of an atom or amino-acid residue corresponds to the degree of solvent accessibility.

To carefully analyze the difference, we used the *PISA* server (https://www.ebi.ac.uk/pdbe/pisa/) and *BIOVIA Discovery Studio*. The interface area was similar in the heterotetramer and the heterodimers, but the heterotetramer had more interactions compared with the heterodimers, which might result in a lower solvation free energy (Fig. 3 ▸ and Table 3 ▸). In the heterotetramer, Val62–Gly66 formed hydrogen bonds to the same region of the symmetry-related molecule. There were also interactions of the inserted N-terminal region with Val69, Met77, Phe142 and Phe141 of the VASH1 core regions (Fig. 3 ▸ *a*). Other interactions were similarly observed in the heterotetramer and the heterodimer. Phe60, Phe61 and Arg64 participate in interactions (Figs. 3 ▸ *a*, 3 ▸ *b* and 3 ▸ *c*). Notably, PDB entry 6ocf was stabilized by minimal interaction with the VASH1 core regions (Fig. 3 ▸ *c*). Thus, our tetrameric crystal structure revealed an unexpected variability of the N-terminal region and its stabilization through extensive interactions.

### 3.2. MD simulations of VASH1c–SVBP complexes   

**Figure 4:**
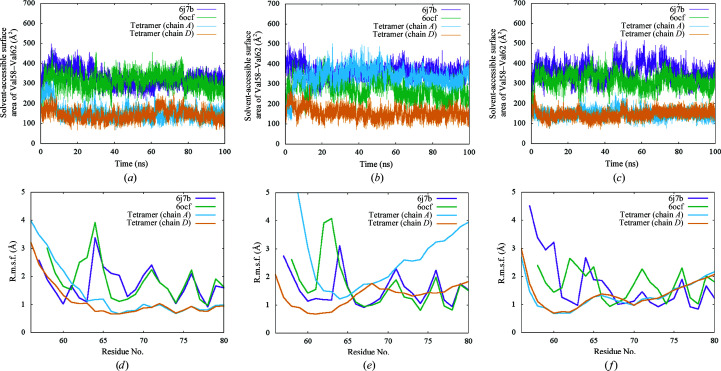
Three independent 100 ns MD simulations of three different VASH1c–SVBP complex structures. (*a*, *b*, *c*) Time evolution of solvent-accessible surface areas of the hydrophobic moiety VPFFV_58–62_. (*d*, *e*, *f*) Root-mean-square fluctuations (r.m.s.f.s) of C_α_ atoms in the N-terminal regions calculated from the MD simulations.

Having found the difference in the N-terminal structure, we performed a 100 ns MD simulation of the heterotetramer and the heterodimer (PDB entries 6j7b and 6ocf) complexes in triplicate to characterize the conformational flexibility and stability of the N-terminus. The C_α_ r.m.s.d.s of VASH1 from the initial structures of the three complexes were less than 3.5 Å throughout the simulation time (Supplementary Fig. S2), indicating that the overall structures of VASH1 are stable for all complexes. VASH1 has five consecutive hydrophobic residues in the N-terminal region, VPFFV at residues 58–62 (VPFFV_58–62_), which contribute to forming the hydrophobic core of VASH1. We calculated the solvent-accessible surface areas (SASA) of VPFFV_58–62_ in chains *A* and *D* of the heterotetramer in PDB entries 6j7b and 6ocf as a function of simulation time (Figs. 4 ▸ *a*, 4 ▸ *b* and 4 ▸ *c*). Interestingly, the SASA values fluctuate around 350 Å^2^ during 100 ns for PDB entries 6j7b and 6ocf. On the other hand, the SASA value of the heterotetramer remained around 150 Å^2^ for 100 ns except for one chain in the second simulation. In this exception, the N-terminus of the VASH1 chain detached from the hydrophobic core of VASH1 at an early stage of the simulation and largely fluctuated throughout the simulation (Supplementary Fig. S3 and Movie S1). When we calculated the r.m.s.f. of C_α_ atoms in the N-terminus of VASH1 in the complexes, the values for the heterotetramer were slightly lower than those for the heterodimers except for the chain in the second simulation, and there were several peaks for the heterodimer which were absent for the heterotetramer. When we analyzed the differences in interaction, the peak at Arg64-Gly65 in the loop region may have been suppressed by the hydrogen bonds that were formed between the backbones of Val62 and Gly66 in the same region of the symmetry-related molecule in the heterotetramer (Fig. 3 ▸ *a*). Two other peaks at Glu71 and Arg76 may have been suppressed by hydrogen bonds between Arg76 and Glu28 of the neighboring SVBP (Fig. 1 ▸ *d*). Additionally, in the heterotetramer we observed that the orientations of the two dimers changes during the simulation (Supplementary Movie S2). As the two dimers are connected mainly through the N-terminal region, there may be a pivotal rotational movement. The results of the MD simulations demonstrate that there is a difference in the flexibility of the N-terminal region between the heterodimer and the heterotetramer.

What are the roles of the N-terminal region (amino acids 56–69) and the significance of the conformational variability? One possibility is that it is necessary for SVBP binding, tubulin binding and cleavage, secretion and activation/inactivation. The previously observed VASH1 processing at Arg76 may be caused by the plasticity of this region (Sonoda *et al.*, 2006 ▸). There are several VASH1–SVBP crystal structures without this region; thus, VASH1 can bind SVBP independently and is not involved in complex formation (Liao *et al.*, 2019 ▸). Moreover, the VASH1 truncation construct (amino acids 77–365) can inhibit neovascular formation, which indicates that the complex is functional and excludes a role in secretion (Sonoda *et al.*, 2006 ▸). It may be that the mobility of the N-terminal region and tetramer formation may negatively control the protease activity of VASH1. Such autoinhibition/processing has been observed for the renin prosegment, which binds and forms a β-sheet with the protease domain to block the active site (Morales *et al.*, 2012 ▸). Interestingly, in the VASH1–SVBP–epoY structure Phe60 of the N-terminal region was close to the epoY inhibitor (Liao *et al.*, 2019 ▸), but in other structures, the same Phe60 side-chain rotamer is oriented differently (Li *et al.*, 2019 ▸). In the VASH2–SVBP–epoY crystal structure, the corresponding Phe49 was also close to epoY (Wang *et al.*, 2019 ▸). In contrast, truncated VASH1 lacking this region was used in the tubulin complex (Liao *et al.*, 2019 ▸), and in the VASH2–SVBP–tubulin complex the side chain of Phe49 was disordered and was not modeled. Moreover, the flexibility of the N-terminal region was partly suppressed by the formation of the heterotetramer, where the neighboring SVBP contacted the region. This conformation might prevent processing at Arg76. As we have observed a pivotal movement of the heterotetramer in MD simulation when the N-terminal region is cleaved, then the VASH1–SVBP complex might be in the fully functional heterodimeric form. Thus, tetramer formation and N-terminal flexibility might lock VASH1 into an inactive conformation preventing substrate binding, catalysis or turnover.

There are several biochemical reports of VASH1–SVBP and VASH2–SVBP complexes. The endogenous full-length VASH1–SVBP and VASH2–SVBP complexes showed tubulin carboxypeptidase activity which was inhibited by alkyne-epoY (Aillaud *et al.*, 2017 ▸) or genetic disruptions (Nieuwenhuis *et al.*, 2017 ▸). The activity of recombinant full-length VASH1 and VASH2 required the presence of SVBP, and the activity was abolished by mutating the catalytic cysteines in VASH1 or VASH2 (Aillaud *et al.*, 2017 ▸; Nieuwenhuis *et al.*, 2017 ▸). Biochemical analysis of the truncated VASH1(52–310)–SVBP complex revealed the enzymatic properties of the VASH1c–SVBP complex, with a *K*_m_ of 7.9 µ*M* and a *k*_cat_ of 44.5 min^−1^ against tubulin peptides (Li *et al.*, 2019 ▸). The difference between the full-length and truncated complexes has not yet been reported. Furthermore, there has been no report of a comparison between the activity of the heterodimer and the heterotetramer. Future biochemical characterization, including that of mutants in the N-terminal region, should reveal the role of the N-terminus in vasohibin function.

## Supplementary Material

PDB reference: VASH1–SVBP complex, 6lpg

Supplementary Figures and captions to Supplementary Movies. DOI: 10.1107/S2059798320011298/ji5016sup1.pdf

Supplementary Movie S1. DOI: 10.1107/S2059798320011298/ji5016sup2.mp4

Supplementary Movie S2. DOI: 10.1107/S2059798320011298/ji5016sup3.mp4

## Figures and Tables

**Table 1:** Data-collection and refinement statistics for the VASH1c–SVBP complex Values in parentheses are for the highest resolution shell.

Wavelength (Å)	1.1
Resolution range (Å)	40.76–2.30 (2.382–2.300)
Space group	*P*6_1_22
*a*, *b*, *c* (Å)	71.854, 71.854, 215.811
α, β, γ (°)	90, 90, 120
Total reflections	298269 (30582)
Unique reflections	15487 (1492)
Multiplicity	19.3 (20.5)
Completeness (%)	99.88 (100.00)
Mean *I*/σ(*I*)	24.93 (2.48)
Wilson *B* factor (Å^2^)	48.41
*R*_merge_	0.09476 (1.336)
*R*_meas_	0.09735 (1.370)
*R*_p.i.m._	0.02208 (0.3012)
CC_1/2_	1 (0.807)
CC*	1 (0.945)
Reflections used in refinement	15473 (1492)
Reflections used for *R*_free_	1548 (149)
*R*_work_	0.1896 (0.2300)
*R*_free_	0.2362 (0.2910)
CC_work_	0.957 (0.874)
CC_free_	0.895 (0.749)
No. of non-H atoms	
Total	2358
Macromolecules	2262
Ligands	20
Solvent	76
No. of protein residues	277
R.m.s.d., bonds (Å)	0.007
R.m.s.d., angles (°)	0.86
Ramachandran favored (%)	95.97
Ramachandran allowed (%)	4.03
Ramachandran outliers (%)	0.00
Rotamer outliers (%)	4.12
Clashscore	6.05
Average *B* factor (Å^2^)	
Overall	60.22
Macromolecules	60.00
Ligands	92.05
Solvent	58.29
No. of TLS groups	10

**Table 2:** Comparison of the torsion angles of the three VASH1–SVBP complex structures

Residue			Tetramer		PDB entry 6j7b		PDB entry 6ocf	
No.	Name	Type	φ (°)	ψ (°)	φ (°)	ψ (°)	φ (°)	ψ (°)
59	Pro	*trans*-Pro	−67.64	131.47	−73.86	151.81	−63.45	148.77
60	Phe	General	−152.24	55.26	−145.14	161.15	−59.22	−35.06
61	Phe	General	−81.68	87.02	−92.34	152.62	−105.38	65.67
62	Val	Ile/Val	−63.70	−19.60	−125.30	132.36	−69.27	−60.18
63	Asn	General	−87.43	−0.80	−74.72	113.22	−41.42	85.62
64	Arg	General	−91.08	127.14	−107.00	8.33	−54.75	174.09
65	Gly	Gly	−80.74	−166.86	−120.12	23.28	79.26	−146.24
66	Gly	Gly	94.78	159.68	75.01	−171.14	−129.36	−142.16
67	Leu	Pre-Pro	−57.58	152.78	−116.39	144.07	−69.55	153.65
68	Pro	*cis*-Pro	−76.18	152.65	−63.54	153.50	−83.35	153.61
69	Val	Ile/Val	−99.93	161.22	−61.19	130.19	−86.70	167.07
70	Asp	General	−68.75	165.91	−66.13	157.87	−74.83	167.19
71	Glu	General	−72.44	−26.88	−55.91	−43.05	−63.08	−44.52

**Table 3:** Interface properties of the N-terminal region of VASH1c with its core region analyzed by the *PISA* server

Structure	N-terminal region	Interface area (Å^2^)	Δ*G* † (kcal mol^−1^)
Tetramer	56–67	551.5	−7.5
PDB entry 6j7b	57–67	659.9	−5.5
PDB entry 6ocf	58–67	540.0	−7.0

† Solvation free-energy gain upon formation of the interface.
