# Supplementary material for: The crystal structure of the tetrameric human vasohibin-1–SVBP complex reveals a variable arm region within the structural core
Source: Acta Crystallogr D Struct Biol. 2020 Sep 16;76(Pt 10):993–1000. doi: 10.1107/S2059798320011298 (PMC7543661; doi:10.1107/S2059798320011298)
Supplement: Supplementary file 1 [file d-76-00993-sup1.pdf]

Acta Cryst D

STRUCTURAL
BIOLOGY

Volume 76 (2020)

Supporting information for article:

The crystal structure of the tetrameric human VASH1–SVBP complex reveals a variable arm region within the structural core

Akihito Ikeda, Seia Urata, Tadashi Ando, Yasuhiro Suzuki, Yasufumi Sato and Tatsuya Nishino

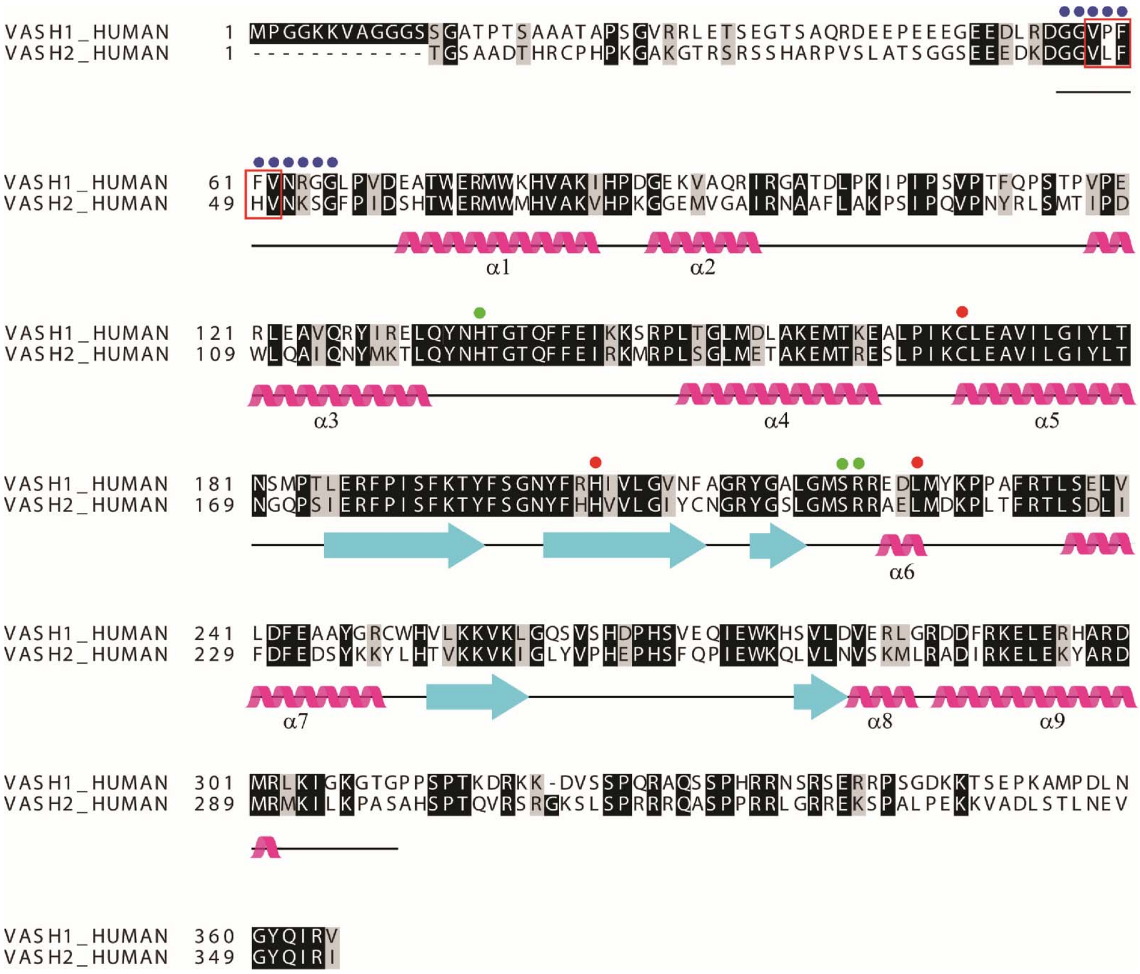

Figure S1 Amino acid sequence alignment of VASH1 and VASH2. The alignment was generated with CLUSTALW and highlighted by BOXSHADE. Catalytic residues are labeled by the red points, amino acids coordinating SO_4^{2-} (Fig. 1C) are labeled by green points, and N-terminal ten residues are labeled by blue points above a letter. N-terminal five consecutive hydrophobic residues VPFFV are marked by the red square.

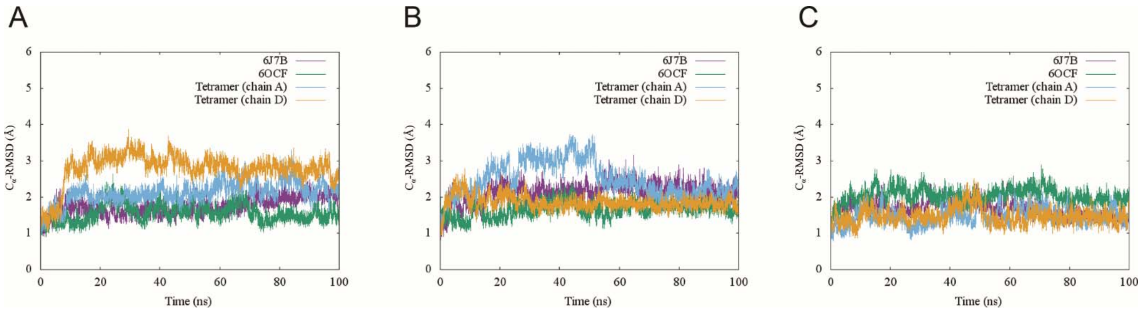

Figure S2 Time evolution of C_α–RMSDs of VASH1c in three independent 100-ns MD simulations of the three diffe66666666666rent complex structures.

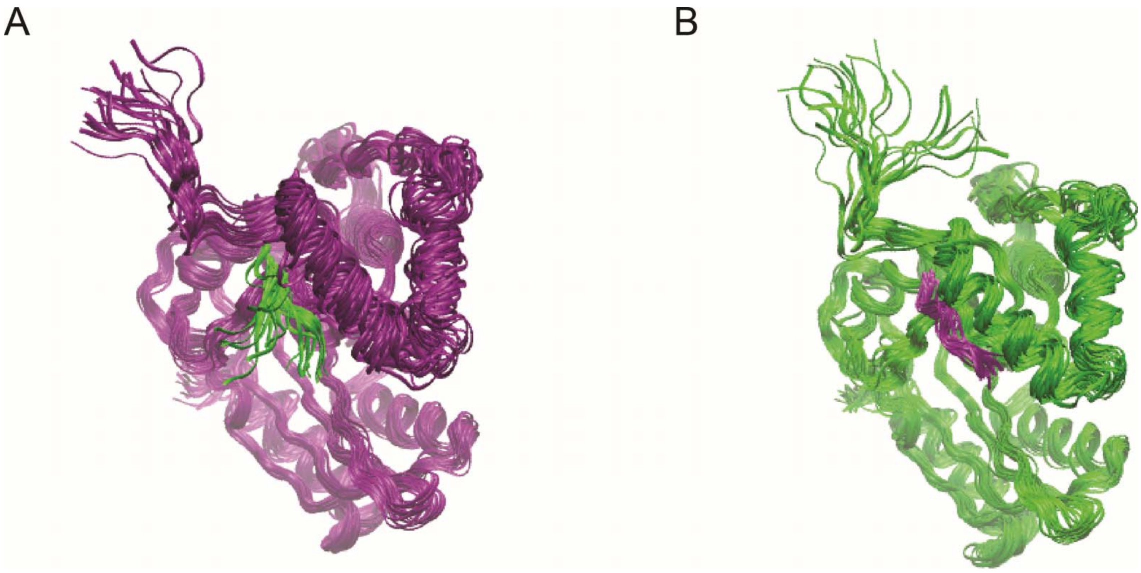

Figure S3 Large fluctuations of the N-terminal region of VASH1c observed in the second simulation of the heterotetramer complex. (A) 20 snapshots of structures of VASH1c/chain D (magenta) and $VPFFV_{58\text{-}62}$ of VASH1c/chain A (green). The structures were superimposed by chain D. (B) 20 snapshots of structures of VASH1/chain A (green) and $VPFFV_{58\text{-}62}$ of VASH1c/chain D (magenta). The structures were superimposed by chain A.

Supplementary Movie S1. Large fluctuations of the N-terminal region of VASH1c observed in the second MD simulation of the heterotetramer complex. Chains A and D of VASH1c are colored by green and magenta, respectively. Chains B and E of SVBP are colored by light green and pink, respectively.

Supplementary Movie S2 Changes in the relative orientation of the two dimers in the heterotetramer complex observed in the MD simulations. Chains A and D of VASH1c are colored by green and magenta, respectively. Chains B and E of SVBP are colored by light green and pink, respectively. The representative trajectory shown in this movie was taken from the first MD simulation. Front view (left) and side view (right) views of the motions of the heterotetramer are shown simultaneously in the movie.
